# The Many Faces of Familial Mediterranean Fever in Children: Lessons From a Moroccan Cohort and Four Diagnostic Pitfalls

**DOI:** 10.7759/cureus.117361

**Published:** 2026-10-02

**Authors:** Mahy Mohamed El Kerim, Anane Sara, Hasnae Elhaddadi, Aziza Elouali, Maria Rkain, Abdeladim Babakhouya

**Affiliations:** 1 Pediatrics, Mohammed VI University Hospital, Oujda, MAR; 2 Pediatrics, Faculty of Medicine and Pharmacy, Mohammed VI University Hospital, Mohammed First University, Oujda, MAR

**Keywords:** colchicine, diagnostic delay, familial mediterranean fever, mefv gene, pediatric autoinflammatory diseases

## Abstract

Introduction: Familial Mediterranean fever (FMF) is the prototypical hereditary autoinflammatory disease. Inherited in an autosomal recessive pattern, it is associated with mutations in the MEditerranean FeVer (MEFV) gene. Its clinical heterogeneity poses a major diagnostic challenge in pediatrics, particularly in developing countries where access to genetic testing remains limited.

Objectives: To describe the clinical, epidemiological, biological, and genetic characteristics of pediatric FMF in the Oriental region of Morocco and to document diagnostic pitfalls through illustrative case reports.

Materials and methods: A retrospective descriptive study including 14 children followed for FMF in the Pediatrics Department of the Mohammed VI University Hospital in Oujda over an 11.5-year period (January 2015 to June 2026). The diagnosis was based on the Yalçınkaya-Özen pediatric criteria and/or genetic confirmation via MEFV gene sequencing.

Results: The mean age at diagnosis was 6.5 years (range: 2-14 years). A marked male predominance was observed in 12 (85.7%); sex ratio (6:1). Parental consanguinity was present in 5/14 (35.7%) of cases. All patients were from the Oriental region 14/14 (100.0%). The predominant clinical manifestations were recurrent fever in 14 (100.0%), abdominal pain in 12/14 (85.7%), and arthralgia/arthritis in 11/14 (78.6%). MEFV gene sequencing was positive in 9/14 (64.3%) of patients, with a predominance of the M694V mutation in 5/14 (35.7%), of whom 3/5 (60.0%) were homozygous. Four illustrative case reports document atypical presentations that resulted in a diagnostic delay of more than two years: a polyarticular form mimicking juvenile idiopathic arthritis (JIA), a coronary aneurysm mistaken for Kawasaki disease, recurrent febrile seizures, and predominantly erysipelas-like erythema. Colchicine was effective in all 14/14 (100.0%) treated cases. No amyloid amyloidosis (AA) was detected during follow-up.

Conclusion: Pediatric FMF is underdiagnosed in the Oriental region of Morocco. Its clinical polymorphism leads to diagnostic delays with harmful consequences. A rigorous clinical evaluation supported by targeted genetic sequencing is essential for early diagnosis and prompt initiation of colchicine therapy.

## Introduction

Familial Mediterranean fever (FMF) is the most common monogenic hereditary autoinflammatory disease in the world [1]. It is the prototypical hereditary periodic syndrome, characterized by recurrent episodes of systemic inflammation and serositis that resolve spontaneously, in the absence of autoantibodies or autoreactive T cells [1]. This autosomal recessive genetic disorder primarily affects populations in the Mediterranean basin: Arabs, Armenians, Turks, Sephardic Jews, and North African populations [2,3]. Its prevalence is estimated to be between one in 1,000 and one in 3,000 in at-risk populations, with a frequency of heterozygous carriers reaching as high as one in five in certain ethnic groups [2,3].

It is caused by pathogenic mutations in the MEditerranean FeVer (MEFV) gene, located on chromosome 16p13.3, which encodes pyrin (marenostrin), a key regulator of innate immunity and inflammasome activation [4,5]. Pyrin dysfunction leads to uncontrolled secretion of interleukin-1 beta (IL-1β), triggering systemic inflammatory flare-ups [6]. Clinically, the disease presents as paroxysmal episodes lasting 24 to 72 hours, characterized by high fever, aseptic peritonitis, pleurisy, synovitis, and, more rarely, a pathognomonic skin lesion resembling erysipelas (erysipelas-like erythema) [7]. The disease begins in childhood in more than 90% of patients [7]. The most serious long-term complication is amyloid amyloidosis (AA)-type systemic amyloidosis, which is effectively prevented by daily, continuous administration of colchicine [8,9].

In Morocco, pediatric epidemiological data remain scarce and are limited to a few national hospital series, notably in Fez and Rabat [10]. To date, no specific published data are available for the Oriental region. In this context, the present study aimed to describe the epidemiological, clinical, biological, and genetic profile of pediatric FMF in Morocco’s Oriental region, while highlighting diagnostic pitfalls and treatment outcomes.

## Materials and methods

Study design and setting

We conducted a retrospective descriptive study over an 11.5-year period, from January 2015 to June 2026, in the Department of Pediatrics at Mohammed VI University Hospital of Oujda, a tertiary-care academic referral center covering the Oriental region of Morocco.

Inclusion criteria

Patients were included if they met all of the following criteria: age between 0 and 16 years at the time of diagnosis, diagnosis of FMF validated by the Yalçınkaya-Özen pediatric criteria [11], and/or confirmation by MEFV gene molecular sequencing; and a minimum of one documented follow-up consultation in the pediatric department.

Exclusion criteria

Patients were excluded if they had incomplete medical records missing critical clinical or laboratory parameters, were lost to follow-up prior to diagnostic validation, or had an alternative hereditary autoinflammatory disease without confirmed FMF criteria or MEFV variants.

Data collection and analysis

Data were collected retrospectively from hospital medical records, hospitalization reports, and the results of laboratory, genetic, and radiological tests. A standardized data collection form was used for each patient, including sociodemographic, anamnestic, clinical, paraclinical, genetic, therapeutic, and follow-up data. The data were entered and analyzed using SPSS version 26 (IBM Corp., Armonk, NY). Qualitative variables were expressed as absolute frequencies and percentages. Quantitative variables were expressed as mean ± standard deviation or as median with range, depending on the distribution.

Genetic analysis

MEFV gene sequencing was performed using targeted next-generation sequencing (NGS) covering the coding exons of the MEFV gene.

## Results

Epidemiological profile

Fourteen pediatric patients meeting the inclusion criteria were identified. All patients originated from the Oriental region of Morocco 14/14 (100.0%). Parental consanguinity was documented in 5/14 (35.7%) of cases, and a family history of recurrent autoinflammatory symptoms was noted in 6/14 (42.9%). The mean age at diagnosis was 6.5 years (range: 2-14 years). The two- to five-year age group was most represented, 7/14 (50.0%), followed by >10 years, 5/14 (35.7%), and 5-10 years, 2/14 (14.3%). No cases were diagnosed under two years of age. A strong male predominance was observed, with 12/14 (85.7%) being male, yielding a male-to-female sex ratio of 6:1 (Table 1).

**Table 1:** Epidemiological and demographic characteristics of the cohort.

Parameter	n (%) or value
Total number of patients	14
Study period	January 2015-June 2026 (11.5 years)
Mean age at diagnosis (years)	6.5 (range: 2-14)
Age group 2-5 years	7 (50.0%)
Age group 5-10 years	2 (14.3%)
Age group >10 years	5 (35.7%)
Male	12 (85.7%)
Female	2 (14.3%)
Sex ratio (male:female)	6:1
Geographic origin (oriental region, Morocco)	14 (100.0%)
Urban setting	9 (64.3%)

Clinical manifestations

All 14 patients fulfilled the Yalçınkaya-Özen pediatric criteria. All 14 cases had recurrent fever (100.0%), followed by paroxysmal abdominal pain due to aseptic peritonitis in 12/14 (85.7%) and inflammatory arthralgia/arthritis in 11/14 (78.6%). Attack duration ranged from one to three days, with an attack frequency of two to six episodes per year. Interictal intervals were completely asymptomatic, with normal inflammatory markers in all patients (Table 2).

**Table 2:** Clinical manifestations in pediatric FMF (N=14). FMF: Familial Mediterranean fever.

Clinical feature	n	Percentage (%)
Recurrent fever	14	100.0
Abdominal pain (aseptic peritonitis)	12	85.7
Arthralgia/arthritis	11	78.6
Asthenia	6	42.9
Weight loss	3	21.4
Vomiting	3	21.4
Erysipelas-like erythema (ELE)	2	14.3
Recurrent febrile seizures	2	14.3
Chest pain (aseptic pleurisy)	1	7.1
Headache	1	7.1

Laboratory and molecular findings

During acute flares, C-reactive protein (CRP) was elevated above 20 mg/L in all 14 (100.0%) patients, with a mean level of 97 mg/L (range: 50-235 mg/L). Neutrophilic leukocytosis was present in 9/14 (64.3%) and mild normocytic/microcytic anemia (Hb < 11 g/dL) in 5/14 (35.7%). Renal function tests (serum creatinine, dipstick proteinuria, 24-hour urinary protein) and autoimmune screens (antinuclear antibodies, rheumatoid factor, anti-neutrophil cytoplasmic antibodies (ANCA)) were negative across the cohort. Molecular analysis of the MEFV gene using targeted next-generation sequencing (NGS) identified pathogenic variants in 9/14 (64.3%) of patients. The exon 10 M694V mutation was the most frequent allele, present in 5/14 (35.7%) of the overall cohort, of whom 3/5 (60.0%) were homozygous (M694V/M694V). The M694I and A744S variants were each detected in 2/14 (14.3%). In 5/14 (35.7%) of patients fulfilling pediatric clinical criteria, standard targeted exon sequencing yielded negative results.

Therapeutic management and evolution

Daily continuous colchicine therapy was introduced in all 14 (100.0%) patients, with age-adjusted dosing (0.5 mg/day for <5 years; 1.0 mg/day for 5-10 years; 1.5 mg/day for >10 years). Complete resolution of attacks was achieved in >50% of cases, and a significant reduction in flare frequency and severity was observed in the remainder, establishing a 14/14 (100.0%) overall clinical response rate. Non-steroidal anti-inflammatory drugs (NSAIDs) were co-prescribed during acute flares in 7/14 (50.0%). Prior to FMF diagnosis, systemic corticosteroid therapy had been inappropriately initiated in 3/14 (21.4%) and intravenous immunoglobulins (IVIG) in 1/14 (7.1%). No patient required anti-IL-1 biotherapy (anakinra/canakinumab). Over a mean follow-up period of 4.2 years, no instances of proteinuria or AA amyloidosis were observed (Table 3).

**Table 3:** Treatment modalities administered (N=14). NSAIDs: non-steroidal anti-inflammatory drugs; FMF: Familial Mediterranean fever.

Treatment modality	n	Percentage (%)
Colchicine (continuous daily regimen)	14	100.0
NSAIDs (symptomatic use during attacks)	7	50.0
Systemic corticosteroids (prior to FMF diagnosis)	3	21.4
Intravenous immunoglobulins (IVIG)	1	7.1
Anti-IL-1 biotherapy (anakinra/canakinumab)	0	0.0

Specific clinical presentations

Four patients were selected for detailed description due to the specific diagnostic challenges encountered.

Case 1: Predominantly Articular Presentation

A 10-year-old boy, born to first-degree consanguineous parents, was referred to our department for recurrent episodes of joint inflammation. The first symptoms appeared around the age of five, in the form of intermittent episodes of inflammatory joint pain. Due to the predominance of joint symptoms and their recurrent nature, juvenile idiopathic arthritis (JIA) was initially suspected. As the condition progressed, the inflammatory episodes became more stereotypical and were associated with extra-articular manifestations. During a particularly symptomatic episode, the child experienced abdominal pain and vomiting, followed by polyarthralgia affecting several joints, notably the knees, elbows, shoulders, and proximal interphalangeal joints. Pain in the scapular region was also reported. Clinical examination during the flare-up revealed an inflammatory joint syndrome involving multiple joints (Figure 1). Laboratory tests performed during the flare-up confirmed a significant inflammatory syndrome, with markedly elevated CRP and erythrocyte sedimentation rate (ESR) (Table 4). Neutrophil-predominant leukocytosis, moderate microcytic anemia, and thrombocytosis were also noted. Immunological testing did not provide evidence supporting a systemic rheumatic disease: rheumatoid factor, antinuclear antibodies, and ANCA were negative.

Calprotectin was also negative. Given the association between recurrent febrile episodes, abdominal manifestations, and recurrent joint symptoms, FMF was ultimately suspected. MEFV gene analysis was positive. Treatment with colchicine was initiated at an age-appropriate dose. Clinical improvement was reported during follow-up, with a reduction in inflammatory episodes.

**Figure 1:**
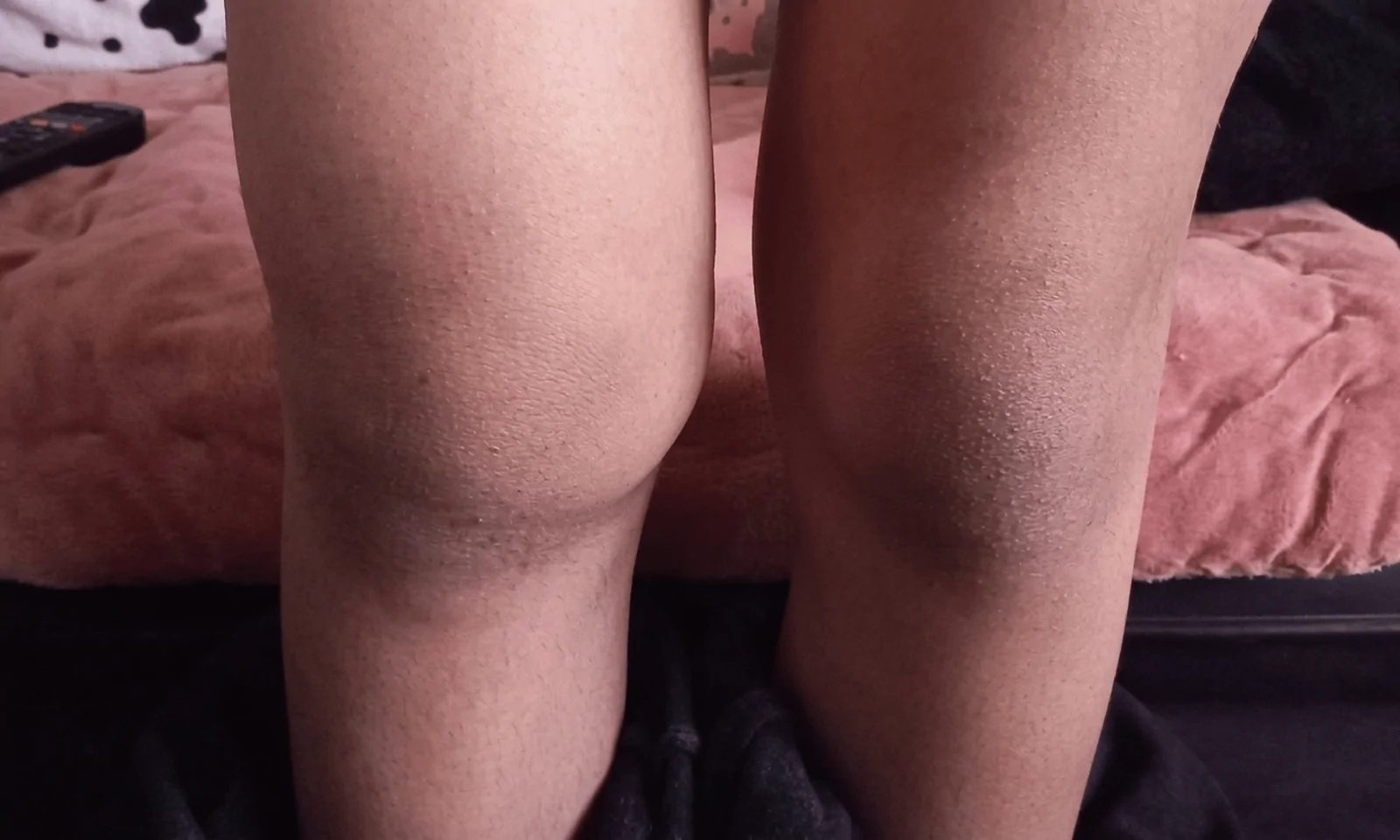
Photo showing swelling of the right knee (arthritis) during a severe attack.

**Table 4:** Laboratory findings in the four illustrative cases. CRP: C-reactive protein; ESR: erythrocyte sedimentation rate; WBC: white blood cell count; NT-proBNP: N-terminal pro-B-type natriuretic peptide; ASLO: antistreptolysin O; RF: rheumatoid factor; ANA: antinuclear antibodies; ANCA: anti-neutrophil cytoplasmic antibodies.

Laboratory parameter	Case 1	Case 2	Case 3	Case 4	Reference range
CRP (mg/L)	105	235	63	83	<5-10 mg/L
ESR (mm/h, first hour)	99	105	90	80	<20 mm/h (age-dep.)
Fibrinogen (g/L)	-	6.8	-	3.6	2.0-4.0 g/L
WBC (/mm³)	16,450	16,080	14,080	8550	4500-13,500 (age-dep.)
Neutrophils (/mm³)	13,900	12,860	10,060	5020	1500-8500
Hemoglobin (g/dL)	10.9	9.3	10.7	11.2	11.5-15.5 g/dL
Platelets (/mm³)	686,000	695,000	470,000	361,000	150,000-450,000
Ferritin (ng/mL)	117	439	-	29	7-140 ng/mL
ASLO (IU/mL)	791	-	-	719	<200 IU/mL
NT-proBNP (pg/mL)	-	354	-	-	<125 pg/mL
Rheumatoid factor/ANCA/ANA	6.7/0/neg	2/0/neg	-	4/0/neg	RF <30 IU/mL; ANA <1:80; ANCA neg.
Calprotectin	0.5	-	-	-	<30 µg/g
Lumbar puncture: (biochemistry/cytology)	-	-	WBC: 3/mm^3^	-	WBC <5/mm³
			Proteinorachia: 0.18 g/l		Proteinorachia: 0.15–0.45 g/l
			Glycorachia: 0.8/serum glucose:1.1 g/l		Glucose: ≥2/3 serum

Case 2: Coronary Artery Aneurysm Mimicking Kawasaki Disease

The patient was a seven-year-old girl born to parents who were second-degree relatives. Her family history included the death of a brother at nine months of age due to suspected vasculitis, with coronary dilation and a severe inflammatory syndrome. For the past two years, she had been experiencing recurrent episodes occurring approximately once a month and lasting two days, characterized by fever, abdominal pain, and vomiting. Twenty-three days before admission, she had developed a fever of 39 °C accompanied by abdominal pain, which persisted for one week. Sixteen days before admission, joint pain in the hip and subsequently in the upper and lower extremities developed, leading to a seven-day hospitalization at a regional hospital prior to her transfer. On admission, she had a fever of 38.3°C, was pale, and was hemodynamically stable. Physical examination revealed painful swelling of the right elbow and bilateral submandibular lymphadenopathy measuring 2 cm. Laboratory results showed a significant inflammatory response, with markedly elevated CRP, ESR, and fibrinogen, leukocytosis with neutrophilia, mild anemia, thrombocytosis, elevated ferritin, and elevated NT-proBNP (Table 4).

Transthoracic echocardiography revealed a 6-mm aneurysm of the left coronary artery (Figure 2). A thoraco-abdominal-pelvic and cerebral CT angiography showed no abnormalities. Kawasaki disease was initially suspected, and the patient received acetylsalicylic acid and intravenous immunoglobulins, resulting in the resolution of fever and joint pain, but the coronary aneurysm persisted. Given the recurrent nature of episodes involving fever, abdominal pain, and joint symptoms, FMF was suspected. Genetic testing of the MEFV gene was positive. Treatment with colchicine was initiated, resulting in good clinical control and a gradual regression of the aneurysm on follow-up ultrasound scans. This case illustrates the diagnostic challenge posed by coronary involvement in an inflammatory context. Although the course of the aneurysm under colchicine treatment is noteworthy, a causal relationship between FMF and coronary involvement cannot be established based on this single observation alone.

**Figure 2:**
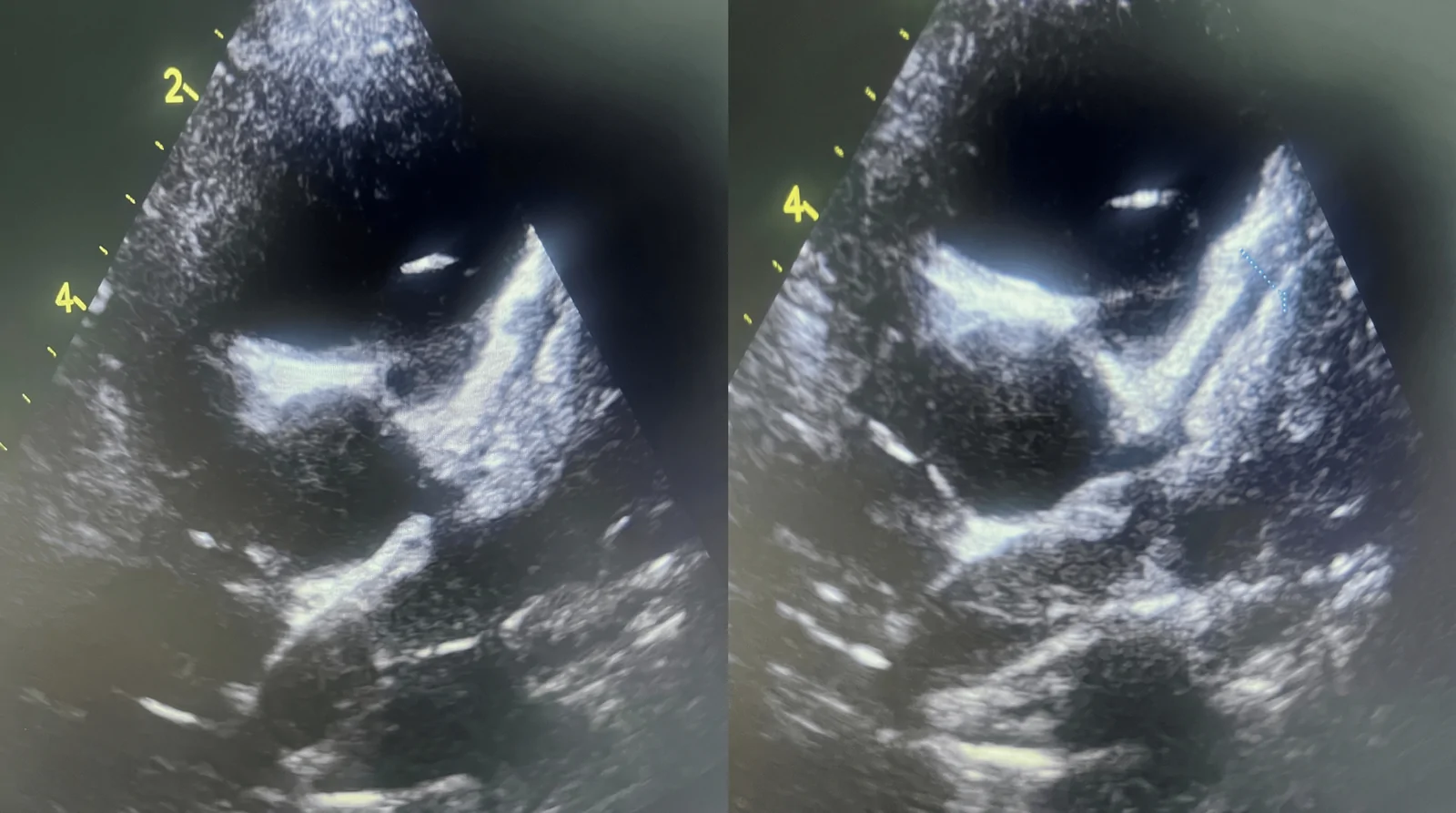
Parasternally oriented short-axis images showing the coronary aneurysm at the time of diagnosis.

Case 3: Recurrent Febrile Seizures

A three-year-old boy born to non-consanguineous parents. He had been hospitalized four times previously for febrile seizures, leading to EEGs (normal) (Figure 3), lumbar punctures (negative), and the initiation of antiepileptic treatment with valproic acid (Depakine®). His personal history included several recurrent episodes (lasting three to five days) characterized by abdominal pain, fever, vomiting, and watery diarrhea. Four days prior to admission, a fever of 39°C developed, which the family did not take seriously (treated with oral acetaminophen). On the day of admission (Day 0), the child experienced a generalized tonic-clonic seizure while febrile, lasting more than 15 minutes. On physical examination, the child had a fever of 38.3°C, was stable, and showed no signs of meningeal syndrome. Watery diarrhea was present.

Laboratory results showed elevated CRP and ESR, leukocytosis with neutrophilia, mild anemia, and thrombocytosis (Table 4). The lumbar puncture was normal (biochemistry and cytology). The brain CT scan showed no abnormalities. Renal and electrolyte function tests were within normal limits. Given the recurrent nature of the febrile episodes with associated abdominal pain and the absence of evidence of a meningeal infection, genetic testing for FMF was initiated and returned a positive result. The patient was started on colchicine and responded well (cessation of attacks), allowing Depakine to be discontinued.

**Figure 3:**
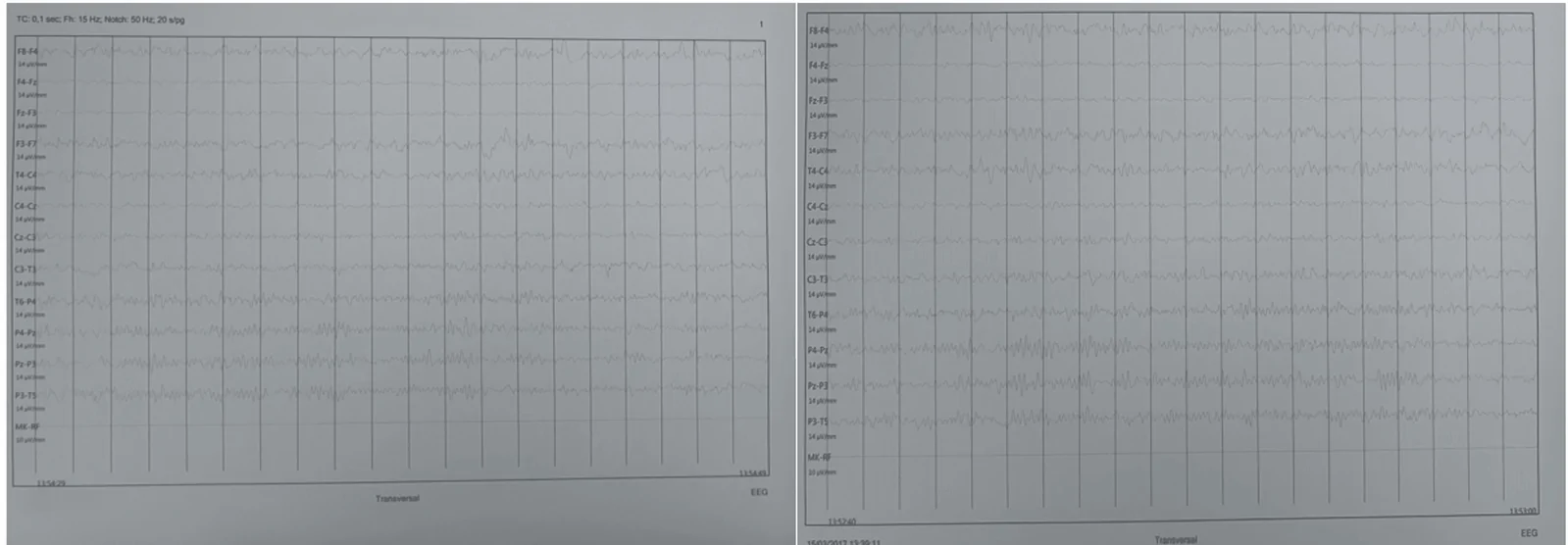
The patient's electroencephalogram during the seizure showed no abnormalities.

Case 4: Predominant Cutaneous Manifestation/Erysipelas-Like Erythema

An 11-year-old girl, the product of a third-degree consanguineous marriage. Symptoms began at age four in the form of joint pain in the lower extremities of an inflammatory nature, transient and migratory, with symptom-free intervals of three to four months. The child had been hospitalized three times prior to diagnosis: once for arthritis of the left ankle associated with a skin rash, a second time for dermohypodermitis of the ankle, and a third time for inflammatory myalgia of the right thigh and knee with a skin rash. This time, the child was admitted for monoarthritis of the left elbow associated with diffuse abdominal pain and a transient erythematous rash on the right lower limb.

On physical examination, the child was afebrile and stable both hemodynamically and respiratorily. The musculoskeletal examination revealed swelling, redness, and warmth of the left elbow, diffuse periumbilical tenderness, and visible dermohypodermitis of the right ankle (Figure 4). Laboratory results showed elevated CRP and ESR, with fibrinogen within normal limits, a mildly elevated white blood cell count, mild anemia, a normal platelet count, and a markedly elevated antistreptolysin O (ASLO) titer; rheumatoid factor and antinuclear antibodies were negative (Table 4). Joint X-rays were unremarkable. Given the recurrent dermohypodermitis (pseudo-erysipelas-like) with polyarthralgia and a clear inflammatory syndrome, FMF was suspected. Genetic testing was initiated and returned a positive result. The patient was placed on colchicine, which effectively controlled the disease.

**Figure 4:**
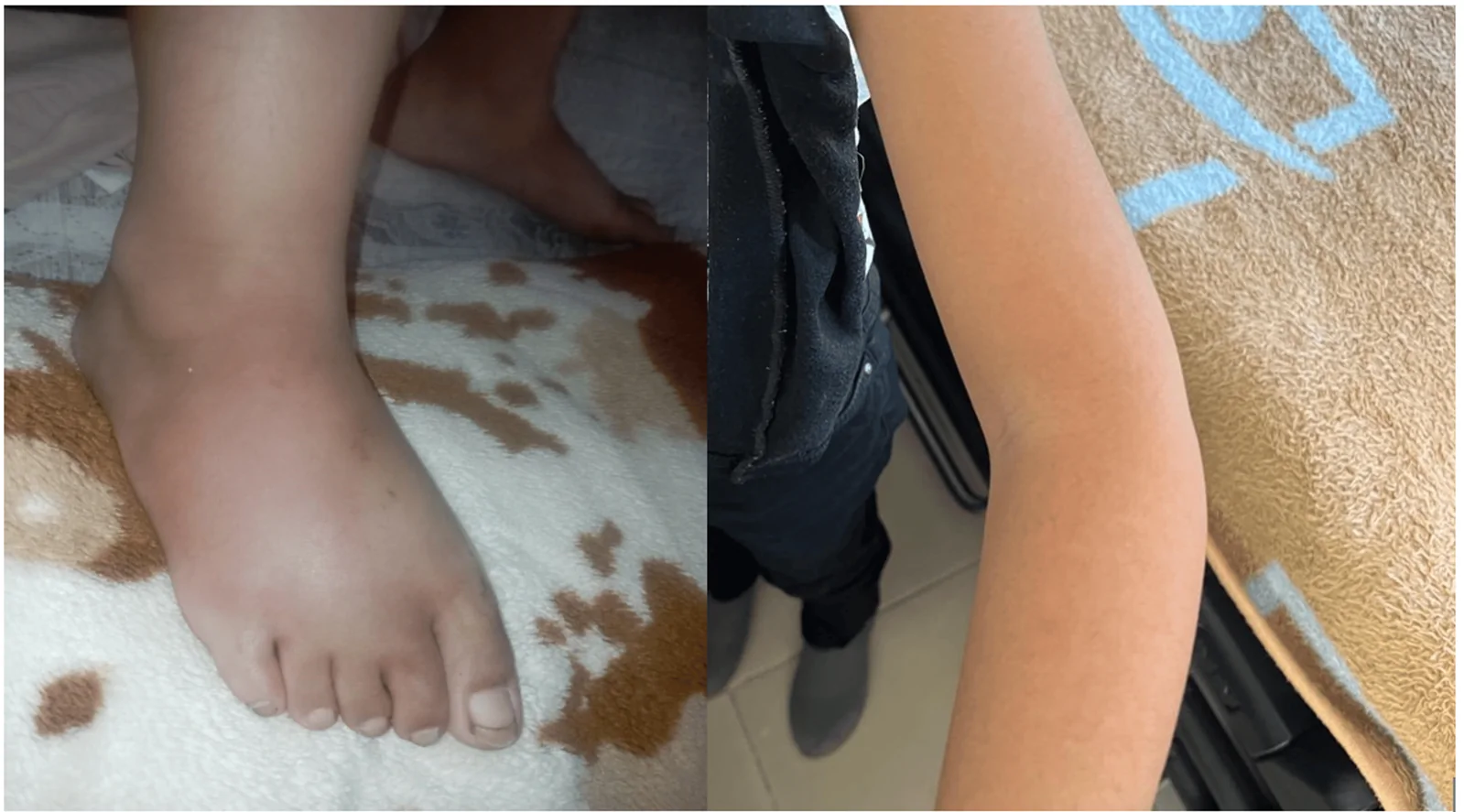
Image showing an erysipelas-like erythema on the right foot and swelling of the left elbow (arthritis).

## Discussion

This study represents the first pediatric cohort describing Familial Mediterranean fever in the Oriental region of Eastern Morocco. Our results demonstrate a mean age at diagnosis of 6.5 years, aligning closely with reported pediatric literature, such as the Fez series by Arraka et al. (7.0 years) and the nationwide Turkish cohort reported by Tunca et al. [10,12]. Interestingly, we observed a striking male predominance (12/14 (85.7%), M/F ratio 6:1), which is significantly higher than figures reported in European cohorts (54% in Turkey, 52% in France) [12]. While FMF is inherited as an autosomal recessive trait without structural sex bias, unequal sex ratios in small regional series may reflect local sociocultural healthcare-seeking patterns or epigenetic modifier loci [13,14]. The parental consanguinity rate in our cohort was 5/14 (35.7%), which is markedly elevated compared to Western European cohorts (<10%), though consistent with North African and Middle Eastern demographic trends [10,14]. High consanguinity increases the likelihood of homozygosity for pathogenic MEFV alleles, particularly the exon 10 M694V mutation, which was identified in 5/14 (35.7%) of our patients (3/5 (60.0%) homozygous). M694V homozygosity is strongly correlated in the literature with early disease onset, a higher frequency of severe arthritis, and an increased risk of AA amyloidosis [9]. 

Clinically, recurrent fever 14/14 (100.0%) and abdominal pain 12/14 (85.7%) dominated the presentation, consistent with global registries [1,12]. However, joint involvement 11/14 (78.6%) was notably higher in our series than in Fez (44%) or Turkey (47%), approaching figures reported in Israeli pediatric series (75%) [15]. This high articular involvement correlates with the high burden of the M694V allele [9,15]. Although none of our patients required escalation beyond colchicine, anti-IL-1 biotherapy (canakinumab) constitutes an effective secondary option in colchicine-resistant pediatric FMF and should be considered when disease control remains inadequate [16]. In our series, we documented four rare, clinically challenging presentations of familial Mediterranean fever (FMF), illustrating the disease's phenotypic heterogeneity and its potential overlap with other pediatric inflammatory conditions.

The first patient presented with recurrent polyarthritis and was initially considered to have JIA. Arthritis is a relatively common musculoskeletal manifestation of FMF and most frequently involves the large joints of the lower extremities [17]. Acute monoarthritis affecting the ankle or knee is the most common pattern, whereas oligoarthritis and polyarthritis, particularly involving the upper extremities and small joints, are less frequently reported [18]. In our patient, the simultaneous involvement of the knees, elbows, shoulders, proximal interphalangeal joints, and scapular regions resulted in an unusual articular phenotype and contributed to the initial diagnostic consideration of polyarticular JIA. The presence of recurrent abdominal symptoms in association with inflammatory joint manifestations was an important additional clue toward an autoinflammatory disorder. This observation emphasizes that FMF should be considered in the differential diagnosis of recurrent or unexplained arthritis in children, particularly when articular episodes occur in association with recurrent abdominal or febrile manifestations.

The second patient presented with prolonged fever, marked systemic inflammation, polyarthritis, and a coronary artery aneurysm detected by transthoracic echocardiography. This clinical presentation initially raised concern for Kawasaki disease. However, the patient did not fulfill the diagnostic criteria considered necessary for Kawasaki disease, and the recurrent history of fever, abdominal symptoms, and inflammatory musculoskeletal manifestations led to consideration of an underlying autoinflammatory disease. The diagnosis of FMF was supported by the clinical phenotype and the identification of an MEFV variant. Cardiovascular involvement in FMF is considered uncommon, although several cardiac manifestations have been reported, including pericarditis, myocarditis, endocarditis, coronary artery involvement, myocardial infarction, and ventricular aneurysms [19]. In addition, several vasculitic disorders have been reported in association with FMF, including Behçet disease, polyarteritis nodosa, and IgA vasculitis [20]. In our patient, the coronary aneurysm occurred in the absence of other documented features of systemic vasculitis. The aneurysm subsequently regressed during follow-up after initiation of colchicine therapy. Nevertheless, the presence of a coronary aneurysm cannot be considered, on the basis of a single observation, as evidence of a specific cardiovascular manifestation of FMF, and a causal relationship between FMF and the coronary lesion cannot be established. The possibility of an overlapping or concomitant inflammatory condition should therefore be considered. The main diagnostic value of this observation lies in demonstrating that an atypical cardiovascular presentation may complicate the recognition of FMF and may initially direct the diagnostic evaluation toward Kawasaki disease.

The third patient presented with recurrent febrile seizures that had initially led to consideration of epilepsy. The clinical history, however, revealed recurrent and stereotyped inflammatory episodes lasting three to five days and characterized by abdominal pain, fever, vomiting, and watery diarrhea. These recurrent episodes raised the possibility of FMF, which was subsequently supported by the clinical phenotype and MEFV genetic findings. Although FMF is a multisystemic autoinflammatory disease, neurological manifestations are uncommon in children. Aseptic meningitis, pseudotumor cerebri, and headaches have been described, including in association with recurrent inflammatory episodes. Epileptic seizures and electroencephalographic abnormalities have also been reported, but the available evidence remains limited and does not establish a causal relationship between FMF and epilepsy or febrile seizures. Ertekin et al. reported two cases of seizures among 52 Turkish children with FMF; one child had a febrile seizure, whereas the other was diagnosed with epilepsy based on electroencephalographic findings [21]. Gedalia and Zamir reported febrile seizures in three Israeli patients with FMF; two experienced seizures during FMF attacks, while the third developed seizures after an episode of aseptic meningitis [22]. In the study by Çomak et al., febrile seizures were reported in 13.2% of patients with FMF, although all seizures were classified as simple, and electroencephalography was therefore not performed routinely [23]. In our patient, no further seizures were reported after initiation of colchicine, and Depakine was subsequently discontinued. However, this temporal association should not be interpreted as evidence that colchicine has a specific anticonvulsant effect or that the seizures were directly caused by FMF. Rather, this observation highlights the importance of considering an underlying autoinflammatory disorder when recurrent fever and gastrointestinal symptoms accompany apparently febrile neurological events.

The fourth patient presented with recurrent inflammatory skin lesions initially diagnosed or considered as dermohypodermitis and associated with recurrent polyarthralgia. The recurrence of these episodes, their association with inflammatory musculoskeletal manifestations, and the absence of supportive immunological findings led to consideration of an erysipelas-like manifestation of FMF. The erysipelas-like erythema is regarded as the characteristic cutaneous manifestation of FMF. It has been reported in approximately 15-20% of children with FMF, although reported frequencies across studies, including patients of different ages, vary considerably, ranging from 3% to 48% [24,25]. The lesions reflect an inflammatory process involving the skin and are associated with neutrophilic inflammation. They are usually painful, erythematous, and localized to the lower extremities, particularly the anterior aspect of the legs below the knee and the dorsal surfaces of the ankles and feet. The lesions are most often unilateral but may occasionally be bilateral and symmetrical [24-26] (Table 5). They are generally self-limited and resolve within approximately 48-72 hours. Physical exertion may precipitate or exacerbate the lesions. In our patient, the recurrent erythematous lesions were associated with inflammatory joint manifestations and abdominal pain, and the diagnosis of FMF was supported by MEFV genetic testing. Treatment with colchicine, together with bed rest and analgesics during the acute episode, resulted in a favorable clinical response and good disease control. This observation emphasizes the importance of recognizing the erysipelas-like presentation of FMF, particularly when recurrent skin lesions are repeatedly interpreted as bacterial dermohypodermitis.

**Table 5:** Comparative analysis of clinical features with national and international cohorts. ELE: Erysipelas-like erythema.

Manifestation	Our study	Arraka et al. (Fez, Morocco) [10]	Tunca et al. (Turkey) [12]	Koné-Paut et al. (France) [26]	Infevers Registry [1]
Recurrent fever	100.0%	100.0%	96.0%	100.0%	97.0%
Abdominal pain	85.7%	88.0%	89.0%	93.0%	88.0%
Arthralgia/arthritis	78.6%	44.0%	47.0%	71.0%	55.0%
Cutaneous (ELE)	14.3%	0.0%	21.0%	18.0%	25.0%
Chest pain	7.1%	0.0%	36.0%	33.0%	30.0%
Colchicine response	100.0%	100.0%	>90.0%	>90.0%	>85.0%

Taken together, these four observations illustrate the clinical heterogeneity of FMF and the diagnostic difficulties that may arise when its manifestations overlap with other pediatric inflammatory disorders. The articular presentation mimicked JIA, the coronary presentation initially raised concern for Kawasaki disease, the recurrent febrile seizures complicated recognition of the underlying autoinflammatory disease, and the cutaneous presentation initially suggested recurrent dermohypodermitis. These cases emphasize that FMF should be considered in children presenting with recurrent, stereotyped inflammatory episodes, particularly when fever is associated with abdominal, articular, or characteristic cutaneous manifestations. At the same time, atypical manifestations should be interpreted cautiously, and genetic findings should always be integrated with the clinical phenotype rather than considered diagnostic in isolation. This approach is particularly important when uncommon cardiovascular or neurological findings are present, as the available evidence may not be sufficient to establish a direct causal relationship with FMF.

Limitations of study

This study has several limitations. First, the small sample size (n=14) from a single tertiary care center limits the statistical power and generalizability of our findings and precludes formal statistical comparison with other cohorts. Second, the retrospective design carries inherent risks of recall and documentation bias. Third, standard targeted exon sequencing failed to identify pathogenic MEFV variants in 35.7% of patients meeting clinical diagnostic criteria, which may reflect incomplete genomic coverage or non-coding regulatory variants not captured by this approach. Finally, the mean follow-up period of 4.2 years may be insufficient to fully exclude the later development of AA amyloidosis, a complication that can manifest after prolonged disease duration.

## Conclusions

Pediatric Familial Mediterranean fever is an underdiagnosed condition in Eastern Morocco characterized by a high rate of consanguinity and M694V mutation prevalence. Its clinical heterogeneity frequently leads to prolonged diagnostic delays and inappropriate therapies. Increased awareness among general pediatricians, early application of validated pediatric diagnostic criteria, and prompt initiation of colchicine are paramount to preventing long-term amyloid complications.
